# Classifying Sleep Slow Oscillations in Low Density EEG

**DOI:** 10.1007/s12021-026-09776-3

**Published:** 2026-04-07

**Authors:** Jeffrey Gaither, Peter White, Sara C. Mednick, Paola Malerba

**Affiliations:** 1 Office of Data Sciences, The Abigail Wexner Research Institute at Nationwide Children’s Hospital, Columbus, USA; 2 The Ohio State University School of Medicine, Columbus, USA; 3 Department of Cognitive Sciences, University of California Irvine, Irvine, USA; 4 Center for Biobehavioral Health, Abigail Wexner Research Institute at Nationwide Children’s Hospital, Columbus, USA

**Keywords:** Slow oscillation, Sleep EEG, SHAP, Machine learning, Slow oscillation type, Slow oscillation classifier

## Abstract

Slow oscillations (SOs, 0.5–1.5 Hz) are electrographic signatures of highly synchronous cortical events contributing to slow wave activity (SWA, 0.5–4 Hz) during sleep. An objective, EEG-manifold-based characterization of SOs could enable clinically actionable investigations on their functional role in cognition and health. In an earlier study, our group presented a data-driven approach showing that SOs group into Global, Frontal, and Local patterns, based on near-simultaneous detection across multiple scalp electrodes. However, this method requires sleep acquisitions with at least 24 head electrodes, limiting its applicability in standard diagnostic polysomnography and at-home studies. Here, we introduce a deterministic classification method that leverages machine learning to identify space-time profiles of SO types in low-density EEG (2–8 electrodes). Using an xgboost architecture trained on data from eight or fewer scalp EEG channels, our gradient-boosted tree model drew from more than 300 features to predict SO class (i.e., space-time profile) with high fidelity. Leveraging the feature-based nature of our classifier, we also used the SHAP feature-importance package to isolate the biophysical properties most characteristic of each SO type. Notably, we found that large amplitudes at central electrodes distinguish Global from Frontal SOs, while large amplitudes at frontal electrodes distinguish both types from Local SOs. Finally, we obtained strong predictions using only a minimal setup – 20 features gathered from only F3-F4-C3-C4. Using as few as four leads, this interpretable, low-density EEG approach enables routine spatiotemporal classification of SOs in clinical and research settings and clarifies the biophysical signatures that distinguish them.

**Supplementary Information:**

The online version contains supplementary material available at 10.1007/s12021-026-09776-3.

Sleep slow oscillations (SOs, 0.5–1.5 Hz) are highly synchronous cortical events, generally understood as large traveling waves, which contribute to slow wave activity (SWA, 0.5–4 Hz). Literature has tied SOs to cortical neurodevelopment (Khazipov & Luhmann, 2006; Neske, 2015; Kurth et al., 2017; Trujillo et al., 2019), synaptic homeostasis (Tononi and Cirelli 2006; Lee et al. 2015a; Fultz et al. 2019), and memory consolidation(Maquet, 2001; Walker & Stickgold, 2006; Diekelmann & Born, 2010; Ngo et al., 2013; Rasch & Born, 2013; Dudai et al., 2015; Staresina et al., 2015; Miyamoto et al., 2017; Mikutta et al., 2019; Bastian et al., 2022). In the sleep EEG of adults, while SOs predominantly travel in an anterior to posterior direction and principally emerge at frontal electrodes, they can appear at any electrode and travel, at least in short lengths, through a varying gradient direction (Massimini et al., 2004). This local/global nature of the organization of SOs on the electrode manifold has recently gained attention, as researchers have connected localized SOs and SWA to overnight synaptic reorganization related to daytime learning(Tononi & Cirelli, 2003; Huber et al., 2008; Piantoni et al., 2015), widespread SOs to glymphatic clearance(Lee et al. 2015b; Levendowski et al., 2019; Fultz et al., 2019), and SO propagation patterns to myelin content and cortical connectivity in early adolescence(Kurth et al., 2017).

To capture the large variability of SO profiles on the scalp, our group has introduced a data-driven analysis approach that identified three separate space-time profiles of SOs – Global, Frontal and Local – using an unsupervised clustering algorithm applied to co-detections at multiple electrodes within a fixed delay (Malerba et al., 2019). Despite operating only on a binary matrix of co-detections, subsequent comparisons among Global, Frontal, and Local SOs showed that these types differed in biophysical properties, emerged differently across light (N2) and deep (N3) non–rapid eye movement (NREM) sleep, and coordinated selectively with sleep spindles (Malerba et al., 2019). Specifically, we found a relatively larger fraction of Local SOs in N3 compared to N2 sleep, and a larger fraction of Global SOs in N2 compared to N3. Global SOs travel fronto-occipitally, whereas other types do not exhibit extended traveling profiles. At frontal and central channels, Global SOs show the largest trough amplitudes, followed by Frontal SOs, with Local SOs showing the shallowest amplitudes. At central locations, Global SOs show the strongest coordination (as phase-amplitude modulation) with sleep spindles.

Follow-up work has reproduced the detection of Global, Frontal, and Local SOs in multiple datasets, and showed that the classification of SO types can be leveraged to develop further understanding of the functional role of SOs in cognition and health (Niknazar et al., 2022; Seok et al., 2022; Alipour et al., 2025). By adapting signal processing measures of directional information flow (Generalized Partial Directed Coherence, GPDC) to the dynamics of SOs, we showed that Global SOs create short-lived windows of opportunity in which information processing among distal regions is enhanced; and that GPDC during Global SOs correlates with improved memory performance (Niknazar et al., 2022). This suggests a mechanistic role for Global SOs, in which they create a substrate to coordinate information processing among distributed cortical regions despite NREM sleep being characterized by reduced functional connectivity (Massimini et al., 2005; Born, 2010; Rasch & Born, 2013; Niethard et al., 2018; Ngo et al., 2020). In clinical populations, different patterns of SO subtypes have been linked with specific clinical profiles. For example, recent work has demonstrated distinct SO patterns in people with hypersomnolence with and without depression, reporting that an overabundance of Frontal SOs paired to a reduction in amplitude of Global SOs at the central location can be tied to hypersomnolence specifically (Alipour et al., 2025). Thus, spatiotemporal analysis of SOs reveals type-specific cognitive associations and provides quantitative markers with translational relevance. Altogether, these findings underscore the importance of profiling the space-time organization of SOs in experimental datasets to clarify links to health and cognition, including research and clinical settings where low-density sleep EEG acquisition is standard.

Our initial approach to identifying space-time profiles of SOs relies on an unsupervised clustering algorithm based on SO co-occurrence across electrodes, allowing three profiles to emerge naturally from the data. This approach currently requires at least 24 head electrodes and about 20 sleep nights of data collection (Malerba et al., 2024). These requirements are often unfeasible in clinical sleep laboratories (at least at scale) and severely limit their deployment in case studies. The unsupervised nature of the method makes it impossible to directly extend the classifications to novel datasets, requiring a re-establishment of the three classes every time. Therefore, to support our goal to allow the community to identify SO space-time dynamics in a wide variety of research contexts, we have developed a machine learning (ML) model that can reliably assign a label of Global, Frontal, or Local to any given SO event with high fidelity and generalizability using only 4–8 electrodes and minimal computational power.

## Materials and Methods

### The Global, Frontal, and Local SOs Datasets

This study relied on two datasets of sleep polysomnography nights acquired in the Mednick Lab at the University of California, Irvine. The first was a base dataset (used to train and test our ML model) comprising of EEG data from 22 healthy individuals (9 females and 13 males; ages 18–34, mean age 21.9) acquired with a 64-channel setup, including 58 head EEG channels sampled at 1000 Hz and six additional channels used for reference, grounding, and the capture of other biosignals such as electromyographic, electrooculographic and electrocardiographic (Seok et al., 2022; Alipour et al., 2024). In accordance with our goal to create a robustly generalizable model, the second dataset was set apart as a validation set of nighttime sleep from 34 healthy adults (18 females and 16 males; ages 18–30, mean age 20.7) acquired with a 32-channel EEG, including 24 head channels sampled at 1000 Hz and 10 channels supporting collection of other biosignals (mastoids, EMG, EOG, EKG) (Rechtschaffen & Kales, 1968; Malerba et al., 2019). In both cohorts, the data was originally referenced through FCz and then re-refenced to the contralateral mastoid; reference was not subsequently adjusted during analysis. The similar age distributions of the two cohorts addressed the well-attested effect of age on SOs (Landolt et al., 1996; Carrier et al., 2001; Mander et al., 2017; Rosinvil et al., 2021) and restricted the applicability of our classifier to persons falling in this age-range. The Mednick laboratory performed sleep staging to divide the data into N2, N3 and additional sleep-stages following the criteria in Rechtschaffen & Kales’ manual as applied in the Matlab package HUME, and identified epochs in which all channels were free of artifacts. Artifact marking was performed first by visual inspection (Mednick group), and supplemented by algorithmic detection in each channel (Malerba group). We used two computational algorithms for removing muscle movement artifacts. In the first, introduced by Brunner and colleagues, we filtered the signal to the range 26.25–32 Hz and then split into 4-second time bins; bins more than 4 times greater than the median of the 45 bins (3 min) surrounding the epoch were excluded as artifacts (Brunner et al., 1996). Second, we applied an analogous method from Wang et al. that used a similar filtration to 4–50 Hz, a splitting of the signal into 5-second bins, and a removal of bins exceeding 6 times the median value over all bins (Wang et al., 2020).

We detected SO events at each channel for all nights in both datasets, leveraging the algorithmic detection approach developed by our group (Niknazar et al., 2022; Alipour et al., 2025), which closely follows those set forth in Massimini, and in Dang-Vu (Massimini et al., 2004; Dang-Vu and others, 2008). In brief, the signal was band-filtered to the range 0.1–4 Hz, and an SO defined to be any segment satisfying the following conditions. First, the event had to be included in the time between three subsequent zero-crossings, with the signal being negative between the first two crossings and positive between the second and third crossings. Also, the first two crossings had to occur between 0.3 and 1s of each other and the whole event (from first to third zero crossing) could span at most 10s. The two time constraints ensured that the de-polarization, hyper-polarization and subsequent relaxation of voltage in the channel were all part of a single physiological event. Second, the trough voltage value was required to be at most − 80 uV (i.e., being a negative value that exceeded − 80), and the whole event range from peak-to-trough had to be at least 80 uV. These requirements ensure that the magnitude of the SO event would be large compared to ongoing dynamics. Among all the candidate events that satisfied the conditions, only events that began and ended within the same sleep stage (N2 or N3) were kept. Lastly, events with amplitudes larger than four standard deviations among the remaining candidate detections at each channel were discarded as possible artifacts.

Our SO sets were subsequently assigned to the categories of Global, Frontal, or Local by applying a k-means clustering to a binary matrix encoding the co-detections of trough events across multiple channels at a short delay (± 400 ms) (Malerba et al., 2019; Niknazar et al., 2022; Alipour et al., 2025; Snedden et al., 2026). Finally, we restricted our study to SOs detected in at least one of our 8 channels of interest (see below). This procedure yielded 118,308 SOs during N3 sleep in our base set (44,477 Global, 34,840 Frontal and 38,991 Local; and mean 5,378 SOs per individual), and 196,131 SOs during N3 in our validation set (86,278 Global, 48,021 Frontal and 61,832 Local; and mean 5,768 SOs per individual). Supplementary Table S1 in the Online Resource provides aggregate SO counts by type across sleep stages and datasets, and Supplementary Table S2 gives SO counts by individual. All these data processing steps were accomplished with in-house software in Matlab (MathWorks, Natick, MA).

Intuitively, these three SO types can be understood as follows: Global SOs as events large in amplitude and showing a large footprint on the scalp; Frontal SOs as moderate amplitude events restricted to mostly the frontal regions; and Local SOs as small amplitude events found in any scalp area without specific preference .

### Extracting the Low-Density EEG Data Related to each SO Event and Defining Features for Classification

**Fig. 1:**
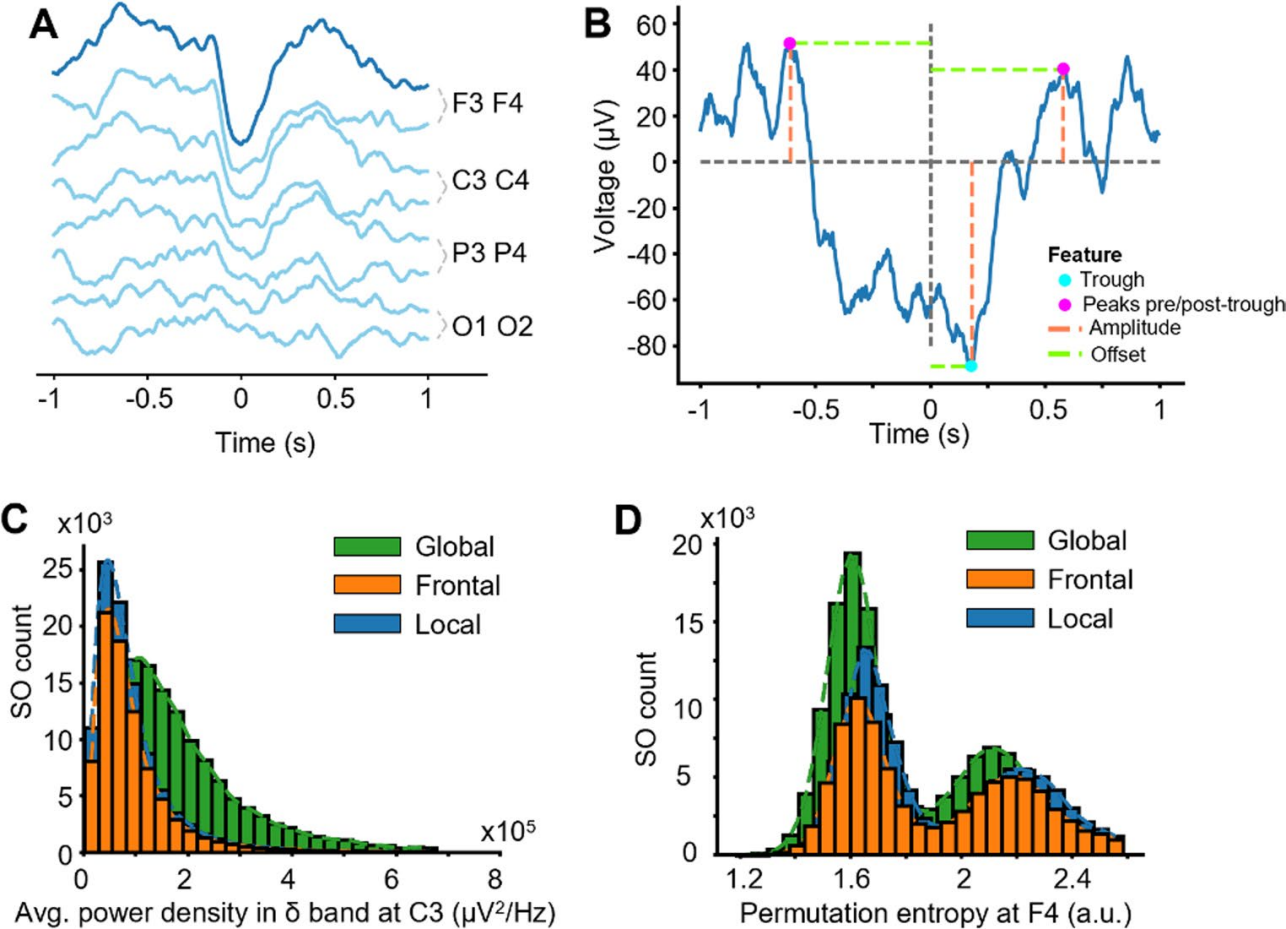
Raw waveform of an SO, with distributions of some selected features. **A** Representative example of the stacked 8-channel EEG during a Frontal SO detected at channel F3, showing 2s surrounding the SO trough. **B**. Plot shows one example of the set of six features used for the ‘base’ model, for a given channel. Three amplitude features are shown in red, while offsets from trough in channel of detection (t=0) appear in green. **C**, **D** Distribution of the values (per SO) of two features across all 314,439 SOs in train/test/validation sets, separated by SO type. Histograms are fit with a smoothing curve to help with intuition. In C, we show average power density in the δ-band at channel C3 (note the broader peak for Global SOs). In D, we show permutational entropy at channel F4 (note bimodal distribution with peaks shifting with SO type). Outliers more than three standard deviations away from the mean were discarded in both plots

Having preprocessed the Global, Frontal, and Local SOs datasets, each SO event was uniquely associated with its channel and time of detection. We then set out to isolate a spatially down-sampled version of the EEG trace for each detected SO. We focused the work on at most 8 channels: F3, F4, C3, C4, P3, P4, O1 and O2, which track activity in respectively the frontal, central, parietal and occipital regions. For each SO event, we exported the data from these 8 channels for a 2s-long epoch centered on the time of the trough of the detected SO. Fig. 1A shows an example SO with the 8 traces stacked from frontal to occipital. We then imported these data into Python as a single large numpy array. For ease of computation, we down-sampled each channel 2s-long epoch to 200 Hz prior to calculating features.

The next step was the computation of ML features, which our classifier would use to infer the SO’s type. We computed 35 total features at each of 8 channels, for a total of 280 predictors. We initially defined a special class of “base features,” chosen for their easy interpretability and high predictive power (Fig. 1B). These base features comprised the amplitude and time offset from the SO detection at three key events: the channel trough, the last upstream peak before the SO’s occurrence (t = 0), and the first downstream peak after the SO’s occurrence. As shown in Fig. 1B, these quantities can be thought of as the x- and y-coordinates of the three events on the graph of the waveform within the channel. We chose these features to be fundamental, robust and accessible. We emphasize that the time-offsets were measured from the node (electrode) of detection and could therefore be very different from 0 in the trough. In addition to the six base features, at each channel we computed 29 additional features, which we refer to as “advanced features.” These values encompass an array of electrophysiological, information-theoretic measures and miscellaneous waveform properties, falling into five categories:

1. *Power* features include the mean power spectrum density value across seven frequency-bands: delta (1–4 Hz), slow-wave activity (0.1–4 Hz), theta (5–8 Hz), alpha (9–12 Hz), sigma (13–15 Hz), beta (16–30 Hz), low gamma (31–48 Hz).
2. *Amplitude* features consist of the mean amplitude of the signal filtered across each frequency-band in the power features, plus the base amplitudes from Fig. 1B, computed with Python’s mne package (Larson et al., 2025).
3. *Time offsets* features are the time offsets shown in Fig. 1B.
4. *Complexity features* span a range of information-theoretic scores computed in Python, including Lempel-Ziv complexity, sign changes, and Hjorth ability, activity and complexity (Cabañero-Gomez et al., 2021), permutation, SVD and sample entropies, Petrosian, Katz and Higuchi fractional dimensions, and detrended fluctuation (Vallat, 2025), and autocorrelation (Harris et al., 2020).
5. *Descriptive* features consist of the node at which the SO was originally detected and the datum of a spindle’s presence (yes/no) computed with the YASA package (Vallat & Walker, 2021).

As an intuitive introduction to the different quantities that the advanced features encode, we plot the distribution of two features in Fig. 1C-D: mean power density in the delta band in electrode C3 and permutational entropy in F4. The mean power density captures the energy of the waveform in a particular frequency band, while permutational entropy quantifies the diversity of ordinal relationships – up, up, down or up, down, up – in the data. The differentiation of feature distributions across the three SO classes is what empowers our classifier. Specifics of the calculation of each feature are given in Supplementary Information, distributions of additional select features are depicted in Supplementary Figure S1, and a full catalog of features with mean values in each channel during N3 is provided in Supplementary Table S3.

### Choice of Multiclass Classifier Architecture, Training Setup

After constructing our bank of feature values for each SO event in each dataset, our next step was to construct classifiers to predict SO type. We began by testing 27 different ML architectures on our train/test dataset using the python package lazypredict (Pandala, 2024) (Supplementary Figure S2), restricting to sleep stage N3 and utilizing all features in a multiclass framework. Importantly, no data from the validation set was used in the construction of the classifiers. The top performing architectures proved to be two tree-based paradigms: LGBMCLassifier and XGBClassifier. Next, because neural nets are often the most powerful architecture but may require meticulous preprocessing, we manually constructed several neural network designs through PyTorch, experimenting with different topologies and input normalization rules. This approach failed to produce a model equaling the performance of our naïve tree-based classifiers. Therefore, we adopted one of the tree-based models as our final architecture of choice, settling on XGBoost for its speed, familiarity and interpretability.

Next, we investigated whether multiple binary classifiers might yield better results than a single multiclassifier. However, multiclass proved the superior option. Finally, we explored whether the Z-normalization of feature-values, either globally or by subject, might confer any gain in performance. No gain from normalization was apparent, so we opted to use raw inputs, a choice that also enhanced the model’s portability.

We employed five random 70 − 30 train-test splits of our train/test dataset, splitting by human subject in order to avoid data leakage between SOs from the same individual. We then trained a model on these five splits, and took our final predicted probability to be the average across the five predictions.

## Results

### Model Performance

**Fig. 2:**
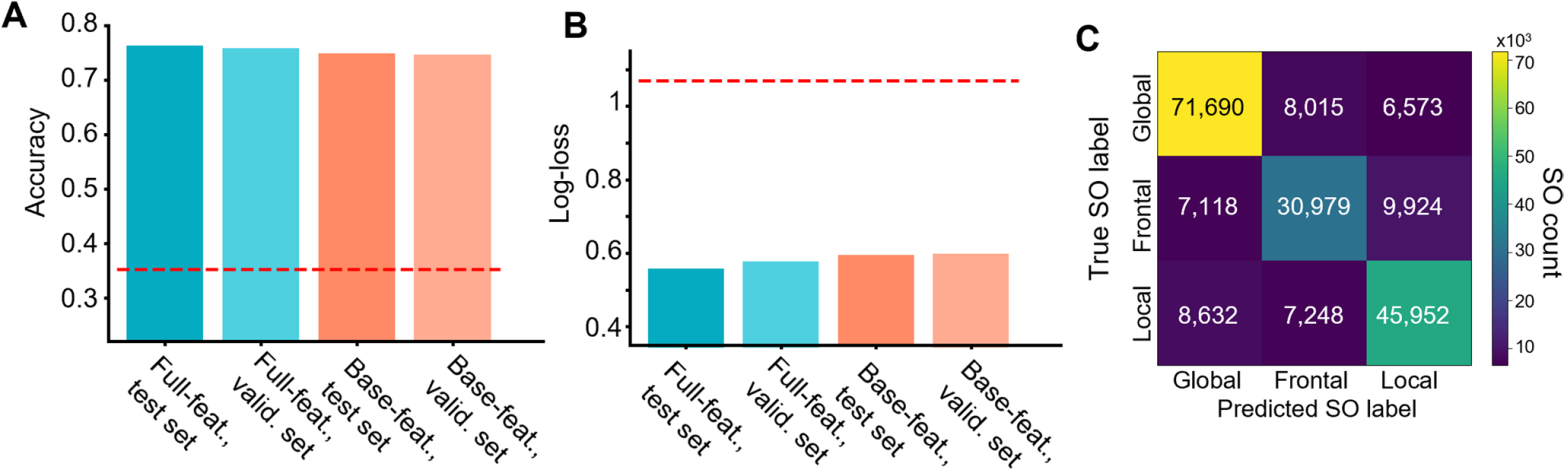
Performance of base- and full-features models in test and validation sets. **A** Accuracy of full- (blue) and base-features (red) models on test and validation sets. Dotted line marks performance of a model that guesses randomly based on SS class proportions. **B** Same as A, but for log-loss. **C** Confusion matrix for the full-features model applied to the validation set

Despite restrictions to a much smaller set of inputs than SO classification has historically utilized, our models achieved high efficacy in identifying the three SO types. Fig. 2 summarizes the performance of our full and base-feature models across our test and validation sets. In Fig. 2A-B, the performance of our full-feature model is shown in blue, while the results for the base-feature model are shown in red. Results on the held-out tests are shown in darker hues, while the lighter hues indicate results on the validation dataset. In both plots, we have included a reference line (in red) for performance at chance. Both our models (base- and full-feature) achieved accuracy (defined as correctly classified records divided by total records (Rainio et al., 2024) between 74% and 77%, with slightly lower performance on the validation set. To verify that accuracy was not achieved solely by trivially assigning every record to the dominant class, we also used the log-loss metric, shown in Fig. 2B. The models showed log-loss values between 0.55 and 0.6, with a slight increase for the validation set.

The confusion matrix in Fig. 2C, which depicts our full-feature model’s performance on the test set, provides a more granular view of the performance. Notably, the most problematic distinction to draw was between the Frontal and Local classes, with the off-diagonal cells, which signify misclassification, containing more elements in this case than do most of the off-diagonal cells involving Global SOs, despite Global being the largest class. We provide confusion-matrix data for all models in Supplementary Table S4.

### SHAP Feature-Importance

**Fig. 3:**
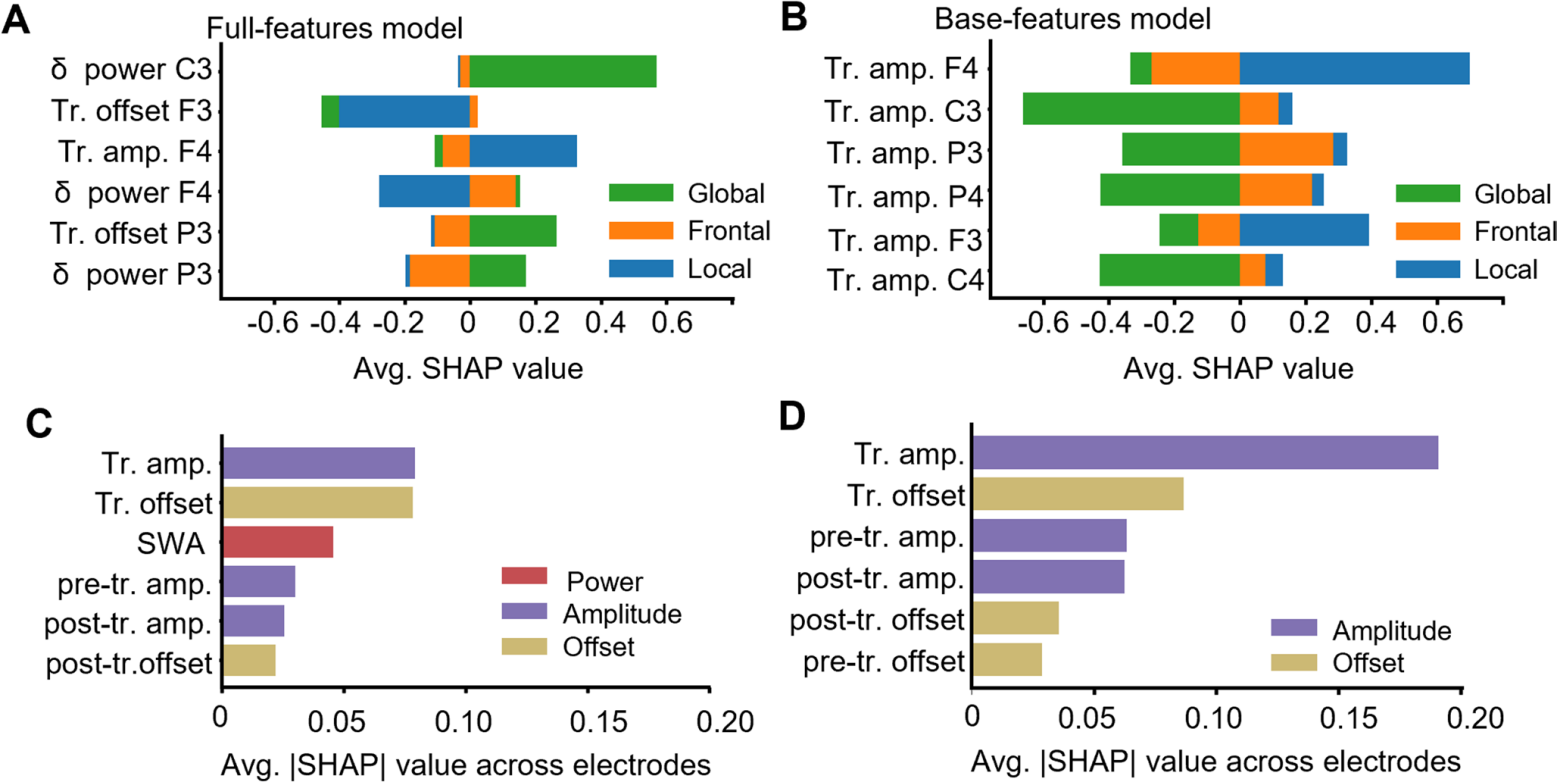
Importance of features for full- and base-feature models. **A** In the full-feature model, average SHAP values for features across three SO types. Magnitude of bar indicates size of effect, and the six features with longest total bar magnitude are shown. Bar direction (positive or negative) matches the correlation between feature values and classification of SO group. Thus, a negative bar simultaneously indicates that low values of the feature associate with the class, and that large values associate against it. **B** Same as A, but for the base-features model. **C** Absolute SHAP value of features averaged across the eight channels and SO events found both in N2 and N3 sleep. **D** Same as C, but for the base-features model

We apply the SHAP feature-importance package to discern the features that are most informative for each SO type (Lundberg and Lee, 2017). These results, computed across each held-out test set, are summarized in Fig. 3. In the plots, we report features ranked by the total length of their SHAP bars. We interpret the top features as being those most characteristic of the three SO types.

In Fig. 3A and B, colors represent the three SO types, while bar length shows the magnitude of the contribution of a given feature to predicting that the SO would be (or not be) of a given type. Bar direction along the x-axis indicates the sign of the correlation between feature and classification. For example, in Fig. 3A, the long right-directed green bar for the first feature of ‘δ-power in C3’ signifies that a high-value of this feature contributes towards a prediction of Global label, and equally, that a low value discourages a Global classification. On the other hand, the long negative blue bar in the second feature, ‘Trough time offset in F3’, indicates that a low value of this feature pushes the model towards a classification of local, while a high value discourages it. Fig. 3A and B depict the top features for our full and base models, as measured by average absolute SHAP value across all classes. The plot shows that the full-feature classifier with 8 channels is likely to use delta power at C3, the offset of the trough at F3 and the amplitude of the trough at F4 when assigning a label to an SO (note that our trough amplitude feature values are kept as negative numbers when passed to the models, so a low value of amplitude means a large trough whereas a large value of amplitude maps to a shallow trough). A more detailed exposition of SHAP and its usage in Fig. 3 is given in the “SHAP Values” section in Supplementary Information, and in Supplementary Figure S3.

All of our features were computed independently for every active electrode. To establish which features were most informative across all electrodes in general, rather than within a particular scalp region, we computed the average absolute SHAP value of each feature across all channels. The results, shown in Fig. 3C and D, show that trough amplitude was consistently the most informative feature on SO type, followed by time offset.

**Fig. 4:**
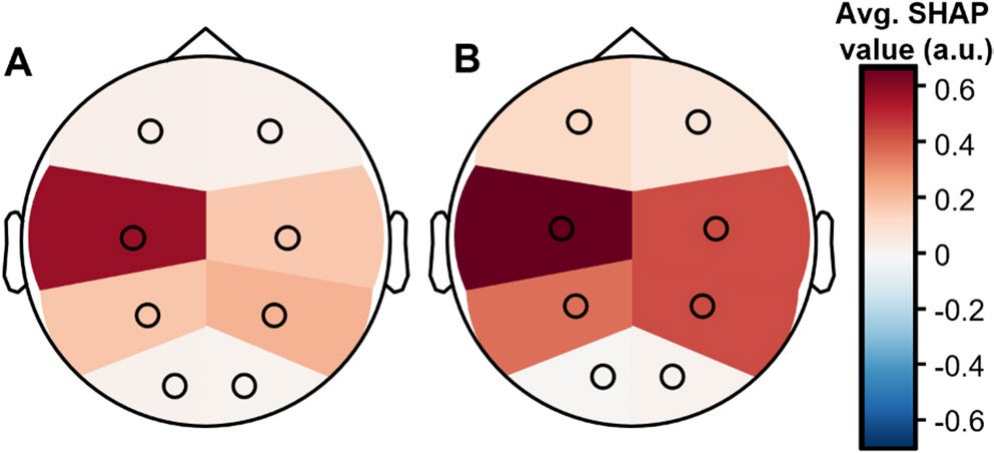
Feature Importance on a topographical representation. Average SHAP value for global SO classification across all channels, for δ power density in a full-feature model (**A**) and negative trough amplitude in a base feature model (**B**)

These results demonstrated that the features of delta power spectral density and trough amplitude were most informative for classification of the full features and base models, respectively. We were interested in establishing whether these features had a differential impact on classification depending on the electrode at which they were measured. Thus, we compared the SHAP value contribution to the Global prediction at each of our eight electrodes on a topographical scalp plot, while using the absolute value for trough amplitude to give large troughs a positive sign and emphasize the similarity of the two features’ substantive contributions. Fig. 4 shows that the two features serve approximately equivalent functions in the two models, with (1) heightened values in central and parietal locations increasing likelihood of global labeling, and low likelihood of frontal or local labeling; (2) large frontal activity in the absence of central activity increases chance of frontal labeling; and (3) frontal activity of average size strongly reduces the likelihood of local labeling.

### Model Performance in Progressively Reduced Sets of Channels

Having demonstrated that we could use feature-derived information from a low-density EEG montage with eight channels to identify the space-time profile of a given SO with high accuracy, we were interested in evaluating whether this approach could achieve comparable success when applied to montages with even lower electrode density. Thus, we re-trained our full- and base-feature models on different channel subsets, progressively removing channels at occipital, parietal and central locations. Our full list of models comprised 1) our original “FCPO” set (eight channels: F3, F4, C3, C4, P3, P4, O1, and O2); 2) an “ FCP” set (six channels, removing the occipital leads); 3) a clinically motivated “FCO” set (six channels, removing the parietal leads); 4) an “FC” set (four channels, removing occipital and parietal leads); and finally 5) a two-channel set “F” (only F3 and F4). Labels for training were derived from the Global, Frontal, and Local SO assigned values using our original algorithm built using a much higher density EEG. This expansion brought our total electrode number of models to 10, with five different sets of electrodes and either the “full” or “base” feature-banks.

**Fig. 5:**
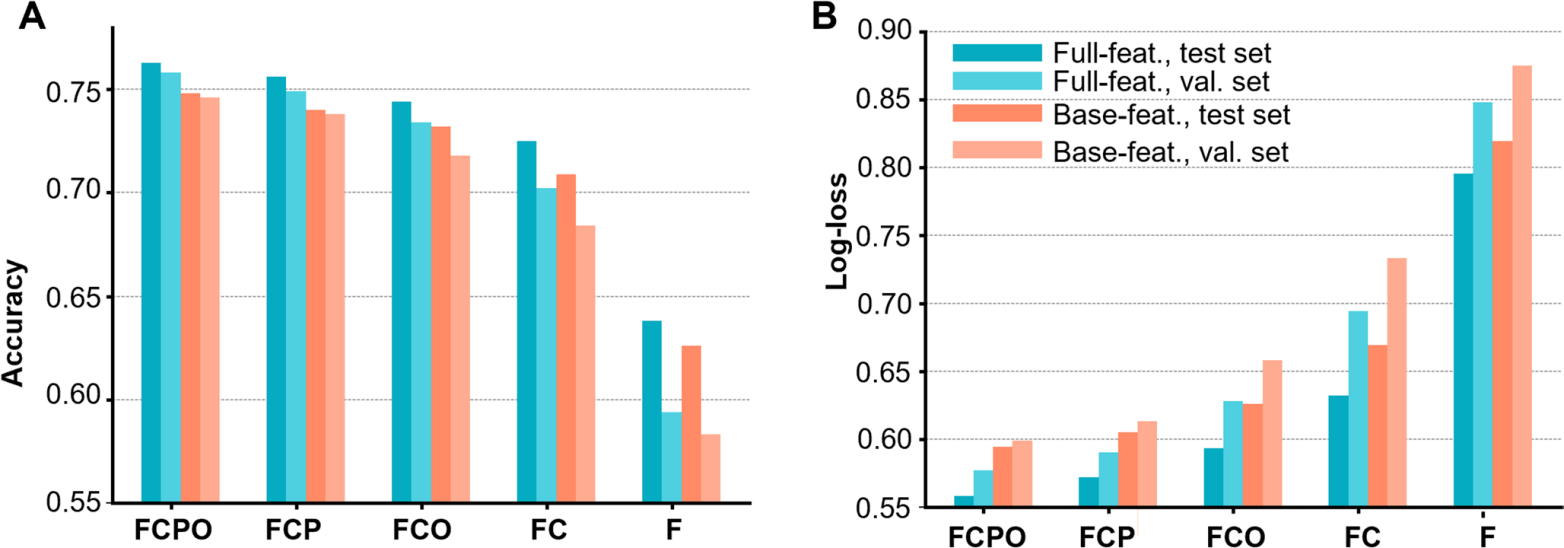
Performance of reduced-channel models on test and validation sets. **A** Accuracy of models leveraging features derived from progressively smaller sets of electrodes. **B** Same as **A** but performance metric of log-loss

We summarize the performance of our full battery of models in Fig. 5. As expected, the models always performed better when leveraging information from additional channels, with the full-feature, 8-channel model achieving the highest test- and validation-set accuracies of respectively 76.3, 75.8%. Nonetheless, the six-node models (FCP and FCO, base or full features) showed only a modest drop in performance, with accuracy remaining over 70% in all scenarios, with relative losses of < 2% for FCP and < 4% for FCO when compared to models of the FCPO set. Furthermore, the FC models (four channels) also showed acceptable performance, averaging around 70% accuracy. We show model-performance under all configurations and datasets in Supplementary Table S5.

### Dependence of Model Performance on Sleep Stage

Having showed that we could achieve classification leveraging SOs that were detected in sleep stage N3, we were curious whether the same approach might be generalizable to other stages. To probe this question, we repeated our entire training and testing procedure on the set of SOs captured during N2 sleep, and then tested both N2- and N3-based models on validation data from both sleep stages.

This study revealed that our models’ efficacy was basically undiminished when trained and tested on N2 rather than N3 data (full results in Supplementary Table S6). Eight-channel models based on N2 achieved accuracy within 1.5% of their N3 counterparts on their corresponding validation sets under both the full- and base-feature frameworks. Similar results held for models built on restricted channel-sets, except the 2-node-model. Furthermore, and somewhat surprisingly, the N3-trained models proved proficient at classifying SOs from the N2 stage, again with a relative loss in accuracy of at worst around 1.5%. However, the N2-trained models were unable to generalize cleanly to N3, with relative accuracy dropping by 7% under most configurations. Thus, our N3 models’ direct portability to novel data from stage N2 indicates a physiology-driven stability of SO attributes across different phases of sleep.

## Discussion

We have built models that accurately identify the space-time profile of an SO using metrics extracted only from a modest number of EEG channels. Our models are fast, straightforward, and demonstrably robust against variation among participants. We also believe them to be generalizable, as the high accuracy of the models was replicated in an orthogonal validation dataset. Our results highlight the features most characteristic of each SO type and thereby provide new insights into SO physiology. Given the SO’s role in episodic memory consolidation, glymphatic clearance, and synaptic homeostasis, our results have implications for the study of cognitive mechanisms in humans and are potentially relevant to translational applications. Furthermore, the established role of SOs in cortical development (Khazipov & Luhmann, 2006; Neske, 2015; Kurth et al., 2017; Trujillo et al., 2019) and observed changes in SOs in children with neurologic disorders (Maski et al., 2015; Kurz et al., 2019; Malerba et al., 2019) motivates closer study of SO space-time types in pediatric populations.

Our most important result is the accuracy, resilience, and generalizability of our models. The level of accuracy achieved by our eight-channel tools, 74–77% in the base and full features models, both in training-test and validation sets, justifies its presentation as a successful fulfillment of our central goal of a portable low-density-EEG-based SO classifier. This performance is especially noteworthy, given the increased difficulty inherent in a multiclass model (i.e., one with more than two outcomes).

Especially striking is our models’ hardly diminished performance when confronted with a novel dataset. Figs. 2 and 5 show that in almost all scenarios, performance on the novel validation set is almost as strong as on the held-out portion of training data. This is not a typical result in machine learning, where models can deteriorate substantially when presented with novel sets. We speculate this superior validation was accomplished through our careful consistency of preprocessing and judiciously chosen set of biologically meaningful features. Also, and most crucially, the physiological properties of the three SO types are evidently robust enough to permit identification in different people. The generalizability of our models achieves our purpose of models that can be confidently deployed in a generic low-density EEG acquisition, consistent with clinical settings.

Portability and interpretability of our models are critically important to their potential translational relevance. Fig. 2 shows that restricting the model’s information to only our “base” features resulted in only a minimal decrease in performance. This robustness allows us to characterize the space-time profiles of SO more transparently. Of note, in Fig. 3C, five of the top six features in our full-features model also belong to our “base” set of features, underscoring the predictive importance of these interpretable and straightforward features of the SO waveform. Another aspect of portability in our models is that they can perform accurately with information derived from progressively lower densities of EEG channels. As expected, our models consistently performed better when provided with additional electrodes (Fig. 5); however, the decline in performance after the removal of each electrode pair was not uniform. The most significant drop in performance occurred in the transition from four to two electrodes, suggesting a special informativeness for electrodes C3 and C4 in establishing the space-time organization of SOs. In contrast, accuracy was fairly consistent between the eight- and six-channel models, and to a lesser extent, between the six- and four-channel models. This again underscores the flexibility and applicability of this approach, and more broadly, of this line of study that centers on recently discovered data-emergent space-time organization of SOs as informative of the role that SO play in cognition and health.

Our work with SHAP values supports an intuitive comprehension of how SO features inform their space-time profiles. The plot showing SHAP values for our eight-channels full-features model (Fig. 3A) suggests that to the model, an SO with high power in the central region is extremely likely to be Global, an SO with high power or large trough in the frontal nodes is either Global or Frontal, and the timing between SO trough at any channel and a local minimum preceding such trough at frontal channels informs on the possibility to be Local (as it speaks to the presence/absence of trough traveling from the front). These properties were shown to be emergent, as evident in the differences in the SO space-time profiles; however, our previous studies could not establish whether these differences would be sufficient to distinguish SOs of different profiles. In fact, our new approach shows that power at central locations, likelihood of traveling from the front, and amplitude at frontal locations are the most characteristic attributes of the Global, Frontal, and Local organization of a given SO, and they are sufficient to establish with some certainty how an SO event is organized on the scalp.

Our full model’s large battery of features – 35 features defined at each of 8 channels – informs on the dependence between numerous traditional and novel metrics and the three SO types, as well as SOs in general. Many metrics in Supplementary Table S3 invite speculation – for example, the fractional dimension and formal complexity of SOs decrease from anterior to posterior locations. However, given the modest improvement of our full-feature over base feature model – only 1–2% in the 8-channel case – the model may be impaired by the inclusion of many uninformative features. However, the xgboost architecture is robust against collinearity and low-content metrics, especially when given copious data on which to train. And our large feature set provides negative as well as positive information as to which features are unimportant to classification of SO type, e.g. the presence of a spindle within one second of SO trough.

We were surprised to see that none of our models leveraged strong information on the presence/absence of a spindle coupled to the SO. Our previous work had shown that, compared to Frontal and Local SOs, Global SOs were most likely to coordinate with spindles at parietal locations (Malerba et al., 2019). However, in our models, features describing the presence/absence of spindles in the time around the SO trough proved class neutral. We interpret this to support that the space-time organization of SOs is an intrinsic property of SO emergence across the brain that is not driven by its coordination with spindle activity. Rather, spindle coordination can be found in SO of any space-time organization and is more likely to occur in the presence of Global SOs. In other words, our results suggest that the machinery that determines if an SO is organized as Global, Frontal, or Local is not the exact mechanism that determines whether a spindle will follow an SO trough at a short delay. In fact, our models trained on SO data from N3 sleep performed almost as well when tested on N2 data, and to a lesser extent the reverse was true (see Supplementary Table S6). This further supports the premise that SO space-time profiles are similarly organized across NREM sleep and further supports the idea that SO space-time organization is established by mechanisms that are different from spindle emergence and coordination mechanisms.

Another noteworthy finding is the laterality bias in features informing on Global SO profiles, which is shown in Fig. 4, and an analogous bias in Frontal and Local SOs (shown in Supplementary Figure S4). For our base-features eight-channels model (Fig. 4B), the size of the trough amplitude in the left hemisphere is more indicative of a Global SO than in the right. Analogously, amplitude in the right forebrain is more typical of Local SOs than amplitude in the left (see Supplementary Figure S4), with analogous trends holding for Global and Frontal SOs. These trends are dampened, although still present, in lighter N2 sleep, although the power-based trends are inverted, probably because of the decreased level of delta power in the N2 stage. Other studies have reported asymmetries of slow-wave sleep, including biases during deep sleep vs. REM sleep (Goldstein et al., 1972) and after acclimatizing to a new location (Tamaki et al., 2016); both these studies found a bias towards the left, consistent with our finding for archetypal Global SOs in Fig. 4B. Another study found the opposed bias, but amongst drug-resistant epilepsy patients (Avvenuti et al., 2020). Precise characterization of these findings lies beyond the scope of this paper, but we replicate them in our validation set (see Supplementary Table S7), confirming their validity.

Our study showed that the most misclassifications consistently occurred between Frontal and Local SOs, across all models except the two frontal-only classifiers, despite Global SO being the largest class (see Supplementary Table S4). This suggests that we did not provide our models with a feature that robustly allowed the disentanglement of the two space-time profiles, at least not to the level of success that models could distinguish Global SOs from the other two profiles. This could be simply due to our choice of features underachieving on this goal, as no study can possibly test all conceivable features. However, in our full-featured models, we expanded the field of considered feature types quite broadly, including measures of entropy, complexity, and permutations, among others. Even if no distinguishing structural property selectively effective to separate Frontal from Local SOs emerged from our analysis, there still is an apparent differentiation of these to space-time profiles in relation to spatial placement, as Frontal SOs are mostly confined in frontal regions, while Local SOs can be emergent at any location. It is possible that a feature embracing multiple regions, such as the quotient of the amplitudes in channels F3/P3, could bridge this gap and increase the model’s power to distinguish between the two types. Additional studies might focus on the distinct interactions of Frontal and Local SOs when detected at the exact locations, to further elucidate if a structural differentiation can be found when removing the focus from Global SOs.

There are limitations to our studies worth discussing. First, our models were trained on data obtained from only 22 individuals and, therefore, may be suspected of yielding inferior performance or insight outside this set. This limitation, however, was largely assuaged by the models’ strong performance on our validation set. Another possible limitation was the exclusion of covariates such as age, sex, and other body metrics (e.g., head size, BMI) from the classifier’s features, a design choice that could have certainly diminished our models’ accuracy. In particular, the effect of age on SO-type distribution and SO characteristics is well-known. However, excluding covariates also increased interpretability and removed the risk of overfitting on such metrics, although it may limit our tool’s applicability to people aged between the late teens and early 30s. Covariates also would have shifted the focus away from the features that characterize the three SO space-time profiles, our central theme, and onto the potentially confounding relationships between the covariates and the three SO profiles. Finally, our training and validation sets were captured by the same research group, albeit with different equipment and at different geographical locations, potentially creating batch effects that our current study cannot verify. Future work will address testing our models with a dataset collected by a different research group, to validate their portability and robustness even more conclusively.

In summary, we have built robust classification models capable of identifying Global, Frontal, and Local SO profiles in sleep EEG densities of eight or fewer electrodes. The performance of our models was excellent in all montages that preserved information from both hemispheres and the central leads. Crucial features of the SO that informed the model’s classification were trough amplitudes, delta power, and time offsets between peaks and troughs. This work enables a transition from a data-driven to a feature-driven approach in establishing the space-time organization of SOs in nighttime recordings, allowing the possibility of studying space-time dynamics in individual nights, and therefore in individual presentations of clinical conditions and/or contextual circumstances.

## Supplementary Information

Supplementary Material 1.

## Data Availability

No new data was generated in this work.
