# Supplementary material for: Classifying Sleep Slow Oscillations in Low Density EEG
Source: Neuroinformatics. 2026 Apr 7;24(2):18. doi: 10.1007/s12021-026-09776-3 (PMC13056764; doi:10.1007/s12021-026-09776-3)
Supplement: Supplementary file 1 — Supplementary Material 1. [file 12021_2026_9776_MOESM1_ESM.pdf]

Supplementary Information: Classifying Sleep Slow Oscillations in low density EEG

Jeffrey Gaither, Peter White, Sara C. Mednick, Paola Malerba

Supplementary methods

Feature Calculation

As described in methods, each SO was encoded in python as an 8 x 401 numpy array, comprising 2s of data sampled at 200Hz from each of 8 channels, with position 201 corresponding to the time of the SO trough in the node of detection. All model features were computed based on this foundational 8 x 401 numpy array, except the identity of the node of detection, which was already known. All feature calculations were performed in Python 3.11.8.

A total of 280 features were computed, 35 on each channel. Each channel was handled separately and independently – there were no inter-channel features.

We first calculated the "base" features for each channel, which depended on three key events in the waveform: the trough and the pre- and post-SO peaks. We defined the trough amplitude to be the minimal amplitude occurring at any timepoint in the 2-second window, and the trough time-offset as the instant at which this amplitude was attained. This definition permitted troughs to occur at the beginning or end of the time-interval. We then detected pre- and post-SO peaks using MNE's peakfinder routine (1.6.1); these were defined as the last (first) peak occurring before (after) the midpoint of the interval, which corresponded to the time of SO detection. If no peaks were reported by mne, we detected the peaks manually as the global maxima closest to the center of the time-interval.

Next we computed Power Spectrum Densities or PSDs for each waveform. We first converted our base numpy array to an mne EpochsArray and then employed the compute_psd() routine. The mean power across each band was computed by first separately restricting the frequency-vector output of compute_psd() to each of our 7 frequency-bands defined in the Methods, and then taking the mean power across the power-vector at the indices corresponding to the band.

To compute Amplitude, we manually filtered our EpochsArray across each band using mne's .filter() routine, which employs a zero-phase FIR filter with a Hamming-windowed sinc, and then calculated mean values amplitude on the resulting numpy array. The Descriptive feature of spindle presence was derived using yasa's spindles_detect routine (0.6.4) with parameters rms=2, duration=.3..1, corr=.4.

The remaining metrics, all of which fell into the Complexity category, were computed using several python packages. The scores LZC, countSignChanges, hjorthActvitiy, hjorthComplexity, hjoirthMobility were all computed with the eeglib package (0.4.1.1). The routines perm_entropy, svd_entropy, sample_entropy, detrended_flucation, petrosian_fd, katz_fd and Higuchi_fd were all run through antropy (0.1.6). Autocorrelation of the signal was computed with numpy (1.26.4).

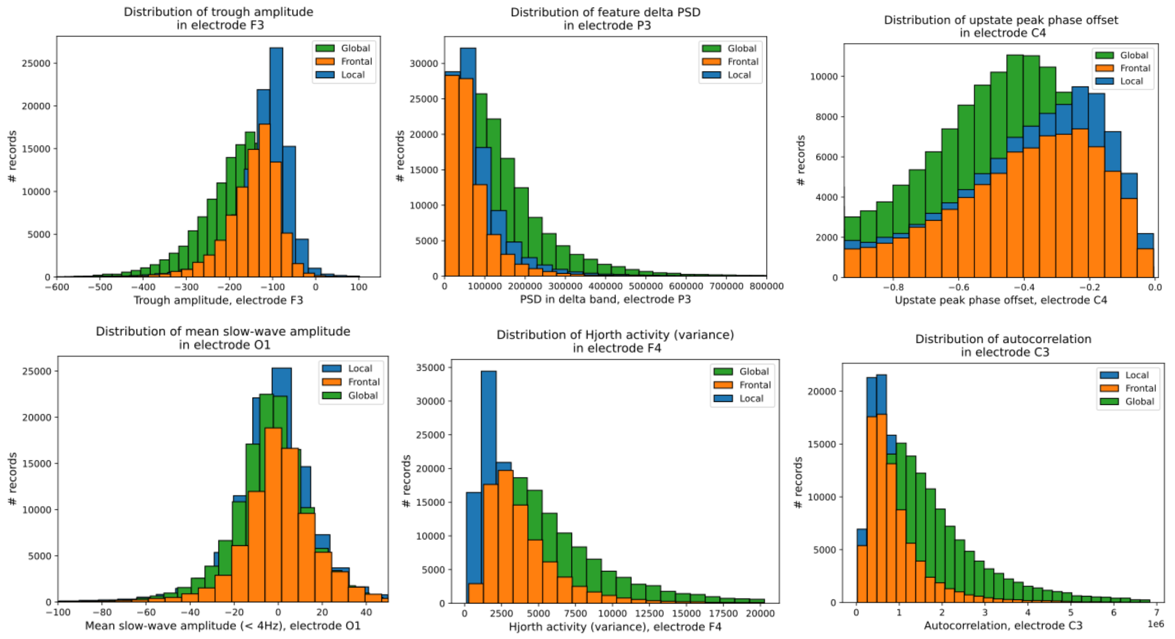

Fig S1. Distribution of feature values for different SO space-time profiles. Distribution of selected features across three SO classes. Plots show feature-values for full set of 314,439 SOs, with outliers more than three standard-deviations from mean removed.

Our purpose in incorporating such a diverse library of features was to optimize performance as well as facilitate biological insight and discovery. However, not all features conferred significant gains to the model. Below in **Supplementary Figure S1**, we depict the distributions of a few representative features across all subjects and N3 epochs.

Model selection, preparation and training

To determine the best ML architecture for our model, we tested 27 separate frameworks using the LazyClassifier routine from the python package lazypredict, with the results shown below in **Supplementary Figure S2**.

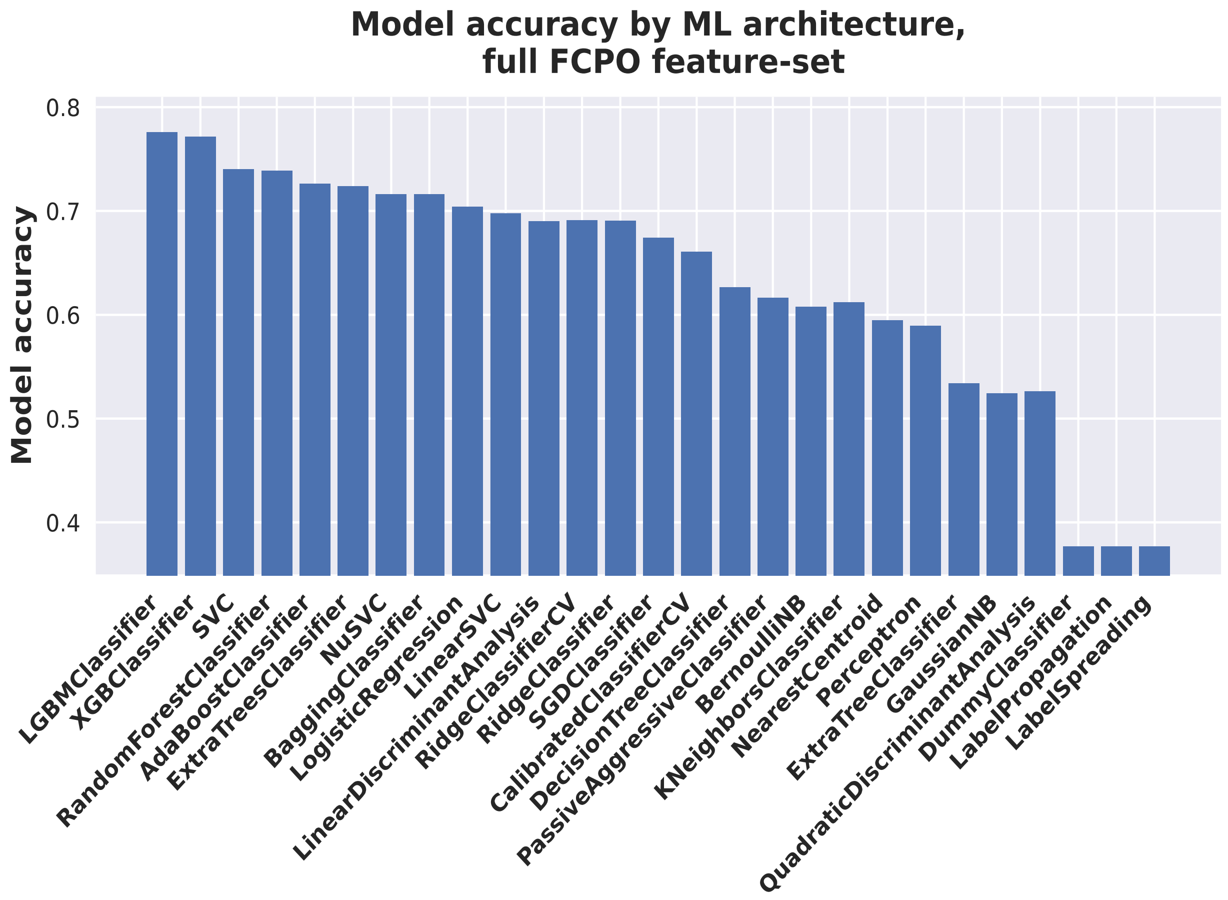

Fig S2: accuracy of each model-architecture on five-fold cross-validated test set, using default parameters. We chose XGBoost due to its performance, familiarity, and easy integration with SHAP.

We ultimately constructed our models in xgboost (2.0.3) with default values for hyperparameters. Training and test subjects for each split were chosen using numpy's random.shuffle routine.

SHAP value

SHAP is a game-theoretic feature-importance assessment tool which computes a feature's contribution as the estimated change in prediction when the feature is removed. A positive shap score indicates that feature pushes the model towards a prediction, while a negative score signifies a pushing away from that prediction.

We summarize the shap values for a given features using an unconventional methodology that accounts for the multiclass nature of our model (3 SO types to identify). Provided the prediction varies consistently with the feature (e.g., the larger the PSD in the delta band of F4, or the smaller the PSD for delta in P3, the more likely to be in a frontal SO) it makes sense to compute the mean shap value for all elements in a given class, and interpret this as the effect of a feature on a prediction. In the binary case, the shap value for the alternate class is redundant, representing only the negative of the value for the positive class.

However, in the multiclass case, a more sophisticated approach is required to sensibly combine the negative contribution to one classification with the positive contribution to another. Using a method employed elsewhere, we summarize a feature's contribution to a prediction as the average shap

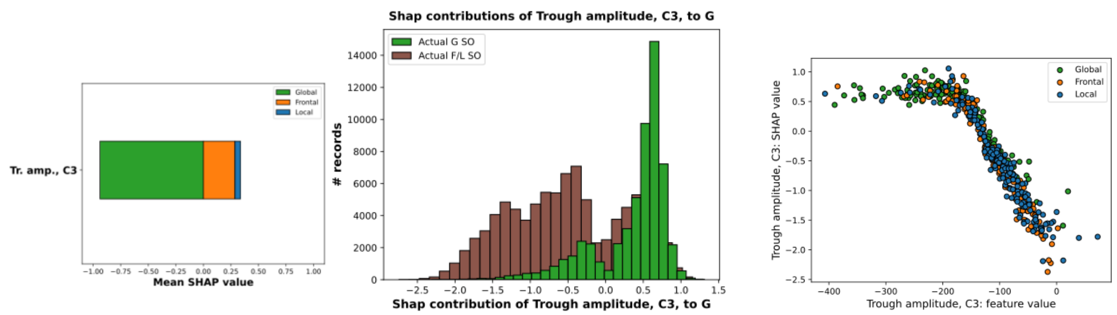

Fig S3. SHAP plots and feature contribution to prediction
S3.A shows a shap plot for feature of trough amplitude in C3. Negative direction of green bar means that negative feature-values contribute towards a classification of "global," while positive values contribute against it. Figure S2.B clarifies this bidirectional correlation with the feature-distributions in the global and non-global cases. Fig S3.C shows correlation between feature and shap-values: though the trend is negative, the relationship is neither linear, nor absolutely accurate in associating global SOs with low feature-values.

absolute value across records, multiplied by the sign of the correlation between the feature and the shap value. Assuming a consistent directionality between feature and prediction, this method captures both positive and negative contributions (read: toward and away from a given prediction) through absolute values.

On this basis, the shap bar can be interpreted as making two separate statements: feature-values in the direction of the shap bar increase the likelihood of a classification, while feature-values in the opposite direction decrease that likelihood. In contrast to the binary case, these contributions are not symmetric among the different classes.

Below in **Supplementary Figure S3**, we illustrate this schematic for the feature 'C3 trough amplitude' in the eight node, base-features model. Figure S3 shows the essential contribution of the feature to classification: a low value (a deep trough) disposes the model towards a verdict of G, while high values lead to predictions of frontal, and very weakly, L. The bars also work negatively – e.g. a high feature-value works against a global classification. The shap distributions for positive and negative classes (from the perspective of a fixed class global) are shown in Fig S3b. Fig S3c shows the negative correlation between the feature and its shap-values in the global class, explaining the negative direction of the green bar in S3a

Topographic SHAP Plots

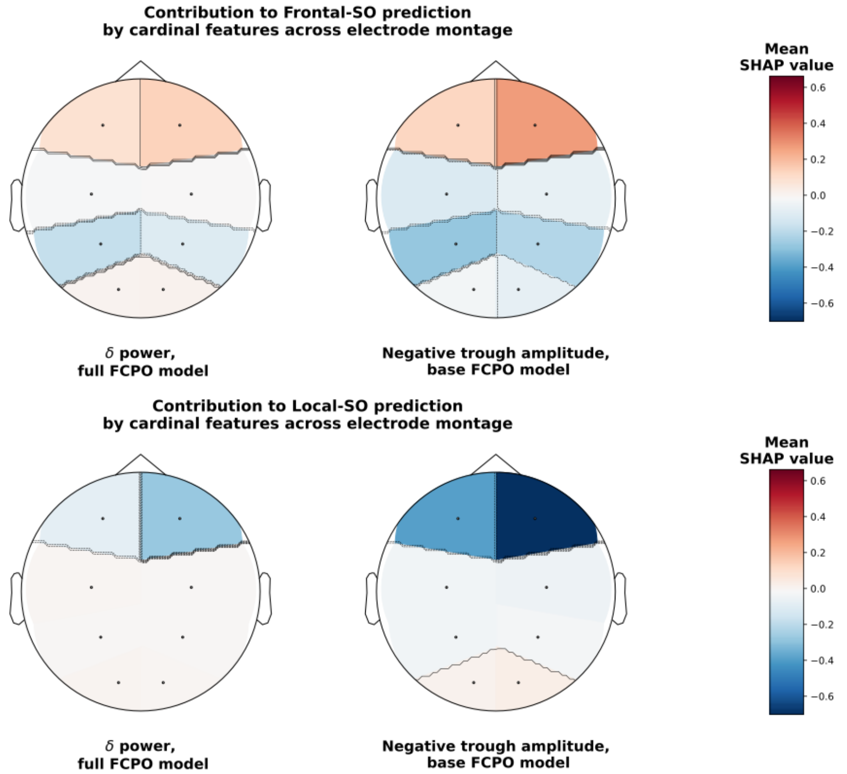

Fig S4: Contribution of cardinal features to Frontal and Local classification across all 8 channels, full and base models. The right-frontal channel F4 is more informative than the left F3 in all four scenarios – markedly so in the base-feature model, where it emphatically distinguishes between frontals and locals. Trough amplitude is negated to make deep troughs positive and facilitate comparison to delta power.

Table S1: SO counts

Dataset	ss	Total	Global	Frontal	Local
traintest	N2	32,911	13021	10901	8989
traintest	N3	118,308	44477	34840	38991
val	N2	82,685	40312	22976	19397
val	N3	196,131	86278	48021	61832

Table S2: SO counts by Individual

Dataset	Individual ID	N3: total	N3: global	N3: frontal	N3: local	N2: total	N2: global	N2: frontal	N2: local
traintest	1	9856	4941	1980	2935	1573	843	368	362
traintest	2	2759	352	1096	1311	1174	183	532	459
traintest	3	8006	2310	3033	2663	1480	461	547	472

traintest	4	3991	620	1764	1607	2063	878	692	493
traintest	5	7262	2187	2876	2199	2275	1008	563	704
traintest	6	4138	991	1650	1497	1663	503	604	556
traintest	7	1052	197	262	593	633	110	232	291
traintest	8	1901	579	347	975	1328	643	417	268
traintest	9	6353	2358	1741	2254	1739	585	708	446
traintest	10	7161	2842	2239	2080	1943	966	548	429
traintest	11	3703	1027	1030	1646	1469	408	589	472
traintest	12	469	13	114	342	201	0	67	134
traintest	13	2705	317	671	1717	1333	292	484	557
traintest	14	2165	146	569	1450	980	135	388	457
traintest	15	2300	372	699	1229	859	70	496	293
traintest	16	5648	2398	1600	1650	1340	751	387	202
traintest	17	14533	8099	3111	3323	2092	1210	385	497
traintest	18	10720	4576	3050	3094	2242	1158	618	466
traintest	19	2302	482	1018	802	1046	212	532	302
traintest	20	6053	2346	1690	2017	1999	988	633	378
traintest	21	11675	6076	3338	2261	2503	1368	674	461
traintest	22	3556	1248	962	1346	976	249	437	290
val	1	1888	410	880	598	1378	389	637	352
val	2	6901	2715	2041	2145	1755	942	488	325
val	3	7349	2187	2019	3143	1943	877	440	626
val	4	965	364	302	299	1323	280	794	249
val	5	6922	2924	1307	2691	2624	1433	582	609
val	6	8002	3963	1868	2171	2987	1965	322	700
val	7	4372	1787	984	1601	5747	3552	990	1205
val	8	10062	5096	1853	3113	3193	1424	867	902
val	9	2924	1001	322	1601	2393	1201	598	594
val	10	18475	9676	4409	4390	3399	1924	684	791
val	11	2724	1088	409	1227	2748	1541	609	598
val	12	2196	372	910	914	903	162	588	153
val	13	4762	1313	1636	1813	1237	394	526	317
val	14	4508	2177	524	1807	1790	1139	398	253
val	15	1985	572	696	717	1539	591	647	301
val	16	6783	2899	1762	2122	2750	1368	812	570
val	17	5625	2273	1280	2072	3226	1598	892	736
val	18	6261	2307	1932	2022	2253	913	792	548
val	19	735	366	97	272	906	397	262	247
val	20	3557	1120	1455	982	1231	356	620	255
val	21	11750	5356	1823	4571	2295	1315	327	653
val	22	3090	1063	1037	990	1871	540	903	428
val	23	7249	2462	2032	2755	4443	1636	1608	1199
val	24	4263	1701	724	1838	2995	1643	743	609
val	25	2597	1135	572	890	2962	1652	829	481

val	26	16505	10331	3990	2184	2406	1633	358	415
val	27	2839	1275	618	946	1236	531	372	333
val	28	4254	1846	1045	1363	3216	1484	961	771
val	29	12526	5627	3376	3523	3960	1876	897	1187
val	30	7626	4165	1895	1566	2938	1775	653	510
val	31	3527	1266	954	1307	1776	634	602	540
val	32	1494	328	426	740	1132	421	410	301
val	33	7571	3001	2159	2411	2241	666	681	894
val	34	3844	2112	684	1048	3889	2060	1084	745

Table S3: Features

Feat cat	Base Y/N	Feature	Mean value of feature at electrode across all N3 SOs							
			F3	F4	C3	C4	P3	P4	O1	O2
Complexity	N	LZC	0.221	0.222	0.229	0.228	0.233	0.233	0.246	0.244
Complexity	N	Autocorrrelation	1980000	1910000	1390000	1270000	1170000	1040000	727000	687000
Complexity	N	num sign changes	8.92	9	9.72	9.63	10.2	10.2	11.7	11.4
Complexity	N	detrended fluctuation	1.82	1.82	1.8	1.8	1.78	1.78	1.74	1.74
Descriptive	N	electrode of origin	0.216	0.222	0.136	0.129	0.108	0.1	0.048	0.0412
Complexity	N	Higuchi frac.dim	1.16	1.16	1.18	1.18	1.19	1.19	1.22	1.22
Complexity	N	Hjorth activity	4940	4790	3480	3170	2920	2610	1820	1720
Complexity	N	Hjorth complexity	5.32	5.3	5.55	5.71	5.68	5.71	5.84	5.97
Complexity	N	Hjorth mobility	0.0711	0.0721	0.0751	0.075	0.0777	0.0786	0.0867	0.0862
Complexity	N	Katz frac. dim.	1.53	1.54	1.56	1.56	1.58	1.58	1.63	1.62
Amplitude	Y	Pre-tr. amp.	75.1	73.8	60.5	57.7	57.3	52.5	45.6	46.2
Phase	Y	Pre-tr. offset	-0.456	-0.452	-0.439	-0.439	-0.423	-0.421	-0.393	-0.398
Amplitude	N	Mean amp, Alpha band	-0.00659	-0.00618	-0.00522	-0.00469	-0.00419	-0.00368	-0.00176	-0.00126
Amplitude	N	Mean amp, high Beta band	-0.00129	-0.00123	-0.00113	-0.00111	-0.000969	-0.000935	-0.000417	-0.000362
Amplitude	N	Mean amp, Delta band	2.07	2.03	1.73	1.62	1.44	1.35	0.662	0.543
Amplitude	N	Mean amp, Gamma band	-0.000226	-0.000228	-0.000247	-0.000243	-0.000229	-0.000218	-9.74E-05	-9.11E-05
Amplitude	N	Mean amp, Sigma band	-0.00428	-0.00414	-0.00352	-0.00332	-0.00291	-0.00273	-0.00126	-0.00104
Amplitude	N	Mean amp, SWA band	-0.0583	-0.762	-2.46	-2.85	-1.96	-3.18	-1.62	-0.811
Amplitude	N	Mean amp, Theta band	-0.00518	-0.00446	-0.00416	-0.00318	-0.00349	-0.00276	-0.00173	-0.00103
Complexity	N	Permutation entropy	1.86	1.86	1.9	1.92	1.93	1.93	1.98	1.99
Complexity	N	Petrosian fractional dimension	1.01	1.01	1.01	1.01	1.01	1.01	1.02	1.02
Power	N	Mean power, alpha band	2070	2060	1260	1140	965	905	696	688
Power	N	Mean power, beta band	82.2	107	78.4	79.5	68.3	71.3	56.7	59.8
Power	N	Mean power, delta band	202000	196000	141000	129000	118000	105000	71100	66800
Power	N	Mean power, gamma band	19.7	23.2	18.3	18.9	15.4	15.4	14	14.3
Power	N	Mean power, sigma band	875	915	765	661	692	634	434	408
Power	N	Mean power, SWA band	238000	230000	167000	152000	140000	125000	84800	79800
Power	N	Mean power, theta band	6120	6280	4460	4120	3730	3450	2680	2650
Amplitude	Y	Post-trough amplitude	80.1	78.4	64.3	60.8	60	55	47.4	48.2
Phase	Y	Post-trough time offset	0.46	0.463	0.47	0.476	0.475	0.476	0.432	0.426
Complexity	N	Sample entropy	0.232	0.235	0.256	0.253	0.271	0.272	0.313	0.308
Descriptive	N	Spindle detected	0.0943	0.0921	0.0891	0.0722	0.0878	0.0781	0.0661	0.0623
Complexity	N	SVD Entropy	0.382	0.384	0.403	0.402	0.416	0.418	0.456	0.453
Amplitude	Y	Trough amplitude	-156	-156	-131	-125	-113	-111	-84.1	-79
Phase	Y	Trough time offset	-0.0221	-0.0223	-0.0193	-0.0173	-0.0137	-0.0161	-0.0124	-0.00647

Table S4: Confusion Matrices

Sleep stage	dset	nodeset	featset	True class	Pred G.	Pred. F	Pred. L
N3	val	All	Full	G	71690	8015	6573
N3	val	All	Full	F	7118	30979	9924
N3	val	All	Full	L	8632	7248	45952
N3	val	All	Base	G	68941	10304	7033
N3	val	All	Base	F	5718	32494	9809
N3	val	All	Base	L	7932	9009	44891
N3	val	6	Full	G	64251	7199	4881
N3	val	6	Full	F	7447	29928	8913
N3	val	6	Full	L	8449	7209	37584
N3	val	6	Base	G	60790	10062	5479
N3	val	6	Base	F	5552	31550	9186
N3	val	6	Base	L	6910	8867	37465
N3	val	FCO	Full	G	49744	8076	4476
N3	val	FCO	Full	F	6732	29257	8777
N3	val	FCO	Full	L	6174	6606	33450
N3	val	FCO	Base	G	46309	10802	5185
N3	val	FCO	Base	F	4853	30942	8971
N3	val	FCO	Base	L	4902	8453	32875
N3	val	4	Full	G	39159	9895	3295
N3	val	4	Full	F	6766	28422	7845
N3	val	4	Full	L	4714	7111	25815
N3	val	4	Base	G	35498	13195	3656
N3	val	4	Base	F	4712	30072	8249
N3	val	4	Base	L	3446	8743	25451
N3	val	2	Full	G	13755	10922	1899
N3	val	2	Full	F	6793	20112	6692
N3	val	2	Full	L	1274	6073	15299
N3	val	2	Base	G	10723	13725	2128
N3	val	2	Base	F	4180	22606	6811
N3	val	2	Base	L	628	7094	14924
N2	val	All	Full	G	32019	4078	4215
N2	val	All	Full	F	3377	15765	3834
N2	val	All	Full	L	1175	3454	14768
N2	val	All	Base	G	29878	5947	4487
N2	val	All	Base	F	2436	16509	4031
N2	val	All	Base	L	943	3638	14816
N2	val	6	Full	G	29281	3836	3411
N2	val	6	Full	F	3271	15314	3625
N2	val	6	Full	L	1077	3313	13590

N2	val	6	Base	G	27038	5947	3543
N2	val	6	Base	F	2353	16050	3807
N2	val	6	Base	L	884	3696	13400
N2	val	FCO	Full	G	21304	4415	3012
N2	val	FCO	Full	F	3141	14549	3652
N2	val	FCO	Full	L	777	3021	12118
N2	val	FCO	Base	G	19332	6361	3038
N2	val	FCO	Base	F	2130	15408	3804
N2	val	FCO	Base	L	620	3384	11912
N2	val	4	Full	G	16138	6777	2032
N2	val	4	Full	F	2510	14927	3139
N2	val	4	Full	L	504	3477	10518
N2	val	4	Base	G	14453	8483	2011
N2	val	4	Base	F	1913	15183	3480
N2	val	4	Base	L	397	3535	10567
N2	val	2	Full	G	6814	4731	1091
N2	val	2	Full	F	2753	9530	2102
N2	val	2	Full	L	166	2667	7199
N2	val	2	Base	G	5385	5908	1343
N2	val	2	Base	F	1735	9788	2862
N2	val	2	Base	L	174	2288	7570
N3	test	All	Full	G	60512	6240	5815
N3	test	All	Full	F	7311	34435	10661
N3	test	All	Full	L	5344	8335	45778
N3	test	6	Full	G	54840	6221	4945
N3	test	6	Full	F	7136	34126	10177
N3	test	6	Full	L	4775	8372	39874
N3	test	FCO	Full	G	42525	5878	4112
N3	test	FCO	Full	F	6882	32248	9713
N3	test	FCO	Full	L	3635	7752	35630
N3	test	4	Full	G	36263	6549	3142
N3	test	4	Full	F	7196	31462	9217
N3	test	4	Full	L	2872	7947	29762
N3	test	2	Full	G	11999	8943	1585
N3	test	2	Full	F	5321	22407	6735
N3	test	2	Full	L	724	6724	18446
N3	test	All	Base	G	59508	6661	6398
N3	test	All	Base	F	7633	33034	11740
N3	test	All	Base	L	5585	8464	45408
N3	test	6	Base	G	53857	6811	5338
N3	test	6	Base	F	7454	32709	11276
N3	test	6	Base	L	4784	8605	39632
N3	test	FCO	Base	G	41995	6181	4339

N3	test	FCO	Base	F	7106	31314	10423
N3	test	FCO	Base	L	3930	7796	35291
N3	test	4	Base	G	35108	7389	3457
N3	test	4	Base	F	7366	30607	9902
N3	test	4	Base	L	2831	8191	29559
N3	test	2	Base	G	11933	8746	1848
N3	test	2	Base	F	5770	21646	7047
N3	test	2	Base	L	861	6712	18321
N2	test	All	Full	G	17234	2320	758
N2	test	All	Full	F	2308	11658	3290
N2	test	All	Full	L	979	2269	10430
N2	test	6	Full	G	15908	2245	652
N2	test	6	Full	F	2269	11598	3253
N2	test	6	Full	L	867	2221	9907
N2	test	FCO	Full	G	12300	1820	490
N2	test	FCO	Full	F	2249	10521	3021
N2	test	FCO	Full	L	640	2080	8898
N2	test	4	Full	G	10575	2174	354
N2	test	4	Full	F	2461	10440	2754
N2	test	4	Full	L	513	2106	8316
N2	test	2	Full	G	4141	2050	299
N2	test	2	Full	F	1751	6086	1996
N2	test	2	Full	L	303	1572	5890
N2	test	All	Base	G	16466	2838	1008
N2	test	All	Base	F	2373	10826	4057
N2	test	All	Base	L	980	2349	10349
N2	test	6	Base	G	15028	2962	815
N2	test	6	Base	F	2350	11000	3770
N2	test	6	Base	L	879	2360	9756
N2	test	FCO	Base	G	11635	2289	686
N2	test	FCO	Base	F	2227	9980	3584
N2	test	FCO	Base	L	657	2093	8868
N2	test	4	Base	G	9904	2720	479
N2	test	4	Base	F	2440	9845	3370
N2	test	4	Base	L	518	2266	8151
N2	test	2	Base	G	4108	2030	352
N2	test	2	Base	F	1896	5622	2315
N2	test	2	Base	L	326	1660	5779

Table S5: Model Performance

model_sleepstage	tested_sleepstage	Dataset	nodeset	featset	acc	logloss	prec_global	prec_frontal	prec_local	rec_global	rec_frontal	rec_local	f1_global	f1_frontal	f1_local
N3	N3	Test	All	Full	0.763	0.558	0.827	0.834	0.83	0.703	0.657	0.679	0.735	0.77	0.752
N3	N3	Test	All	Base	0.748	0.594	0.818	0.82	0.819	0.686	0.63	0.657	0.715	0.764	0.738
N3	N3	Test	FCO	Full	0.744	0.593	0.802	0.81	0.806	0.703	0.66	0.681	0.72	0.758	0.739
N3	N3	Test	FCO	Base	0.732	0.626	0.792	0.8	0.796	0.691	0.641	0.665	0.705	0.751	0.727
N3	N3	Test	6	Full	0.756	0.572	0.822	0.831	0.826	0.7	0.663	0.681	0.725	0.752	0.738
N3	N3	Test	6	Base	0.74	0.605	0.815	0.816	0.815	0.68	0.636	0.657	0.705	0.747	0.725
N3	N3	Test	4	Full	0.725	0.632	0.783	0.789	0.786	0.685	0.657	0.671	0.707	0.733	0.72
N3	N3	Test	4	Base	0.709	0.669	0.775	0.764	0.769	0.663	0.639	0.651	0.689	0.728	0.708
N3	N3	Test	2	Full	0.638	0.795	0.665	0.533	0.592	0.589	0.65	0.618	0.689	0.712	0.701
N3	N3	Test	2	Base	0.626	0.819	0.643	0.53	0.581	0.583	0.628	0.605	0.673	0.708	0.69
N3	N3	Validation	All	Full	0.758	0.577	0.82	0.831	0.825	0.67	0.645	0.657	0.736	0.743	0.739
N3	N3	Validation	All	Base	0.746	0.599	0.835	0.799	0.817	0.627	0.677	0.651	0.727	0.726	0.727
N3	N3	Validation	FCO	Full	0.734	0.628	0.794	0.799	0.796	0.666	0.654	0.66	0.716	0.724	0.72
N3	N3	Validation	FCO	Base	0.718	0.658	0.826	0.743	0.783	0.616	0.691	0.652	0.699	0.711	0.705
N3	N3	Validation	6	Full	0.749	0.59	0.802	0.842	0.821	0.675	0.647	0.66	0.732	0.706	0.718
N3	N3	Validation	6	Base	0.738	0.613	0.83	0.796	0.813	0.625	0.682	0.652	0.719	0.704	0.711
N3	N3	Validation	4	Full	0.702	0.694	0.773	0.748	0.76	0.626	0.66	0.643	0.699	0.686	0.692
N3	N3	Validation	4	Base	0.684	0.733	0.813	0.678	0.74	0.578	0.699	0.633	0.681	0.676	0.679
N3	N3	Validation	2	Full	0.594	0.848	0.63	0.518	0.568	0.542	0.599	0.569	0.64	0.676	0.658
N3	N3	Validation	2	Base	0.583	0.875	0.69	0.403	0.509	0.521	0.673	0.587	0.625	0.659	0.642
N2	N2	Test	All	Full	0.767	0.607	0.84	0.848	0.844	0.718	0.676	0.696	0.72	0.763	0.741
N2	N2	Test	All	Base	0.735	0.673	0.831	0.811	0.821	0.676	0.627	0.651	0.671	0.757	0.711
N2	N2	Test	FCO	Full	0.755	0.627	0.81	0.842	0.826	0.73	0.666	0.696	0.717	0.766	0.741
N2	N2	Test	FCO	Base	0.725	0.679	0.801	0.796	0.799	0.695	0.632	0.662	0.675	0.763	0.716
N2	N2	Test	6	Full	0.765	0.611	0.835	0.846	0.841	0.722	0.677	0.699	0.717	0.762	0.739
N2	N2	Test	6	Base	0.731	0.676	0.823	0.799	0.811	0.674	0.643	0.658	0.68	0.751	0.714
N2	N2	Test	4	Full	0.739	0.663	0.781	0.807	0.794	0.709	0.667	0.687	0.728	0.76	0.744
N2	N2	Test	4	Base	0.703	0.724	0.77	0.756	0.763	0.664	0.629	0.646	0.679	0.745	0.711
N2	N2	Test	2	Full	0.669	0.821	0.668	0.638	0.653	0.627	0.619	0.623	0.72	0.759	0.739
N2	N2	Test	2	Base	0.644	0.834	0.649	0.633	0.641	0.604	0.572	0.587	0.684	0.744	0.713
N2	N2	Validation	All	Full	0.757	0.654	0.876	0.794	0.833	0.677	0.686	0.681	0.647	0.761	0.7
N2	N2	Validation	All	Base	0.74	0.694	0.898	0.741	0.812	0.633	0.719	0.673	0.635	0.764	0.693
N2	N2	Validation	FCO	Full	0.727	0.704	0.845	0.741	0.79	0.662	0.682	0.672	0.645	0.761	0.698
N2	N2	Validation	FCO	Base	0.707	0.757	0.875	0.673	0.761	0.613	0.722	0.663	0.635	0.748	0.687
N2	N2	Validation	6	Full	0.758	0.636	0.871	0.802	0.835	0.682	0.69	0.686	0.659	0.756	0.704
N2	N2	Validation	6	Base	0.736	0.687	0.893	0.74	0.809	0.625	0.723	0.67	0.646	0.745	0.692
N2	N2	Validation	4	Full	0.693	0.801	0.843	0.647	0.732	0.593	0.725	0.652	0.67	0.725	0.697
N2	N2	Validation	4	Base	0.67	0.851	0.862	0.579	0.693	0.558	0.738	0.636	0.658	0.729	0.692
N2	N2	Validation	2	Full	0.635	0.85	0.7	0.539	0.609	0.563	0.662	0.609	0.693	0.718	0.705
N2	N2	Validation	2	Base	0.614	0.886	0.738	0.426	0.54	0.544	0.68	0.605	0.643	0.755	0.694
N3	N2	Test	All	Full	0.761	0.579	0.838	0.865	0.851	0.717	0.634	0.673	0.699	0.764	0.73
N3	N2	Test	All	Base	0.737	0.615	0.823	0.851	0.837	0.688	0.597	0.639	0.668	0.742	0.703
N3	N2	Test	FCO	Full	0.749	0.6	0.812	0.842	0.827	0.719	0.652	0.684	0.707	0.762	0.733
N3	N2	Test	FCO	Base	0.718	0.636	0.785	0.82	0.802	0.683	0.608	0.643	0.677	0.738	0.706
N3	N2	Test	6	Full	0.757	0.587	0.836	0.857	0.846	0.718	0.636	0.674	0.691	0.765	0.726
N3	N2	Test	6	Base	0.732	0.625	0.817	0.837	0.827	0.684	0.605	0.643	0.668	0.741	0.703
N3	N2	Test	4	Full	0.732	0.63	0.789	0.818	0.803	0.706	0.633	0.667	0.697	0.764	0.729
N3	N2	Test	4	Base	0.707	0.668	0.768	0.785	0.776	0.669	0.616	0.641	0.684	0.741	0.711
N3	N2	Test	2	Full	0.686	0.699	0.672	0.693	0.682	0.64	0.599	0.619	0.745	0.782	0.763
N3	N2	Test	2	Base	0.662	0.736	0.657	0.666	0.662	0.608	0.59	0.599	0.726	0.743	0.734
N3	N2	Validation	All	Full	0.754	0.618	0.88	0.772	0.822	0.673	0.684	0.679	0.647	0.798	0.715
N3	N2	Validation	All	Base	0.748	0.625	0.867	0.783	0.823	0.653	0.686	0.669	0.657	0.75	0.7
N3	N2	Validation	FCO	Full	0.729	0.671	0.841	0.738	0.786	0.676	0.669	0.673	0.642	0.792	0.709
N3	N2	Validation	FCO	Base	0.719	0.673	0.842	0.727	0.78	0.64	0.695	0.666	0.652	0.738	0.692
N3	N2	Validation	6	Full	0.755	0.614	0.87	0.784	0.825	0.674	0.683	0.678	0.664	0.786	0.72
N3	N2	Validation	6	Base	0.746	0.622	0.866	0.777	0.819	0.645	0.697	0.67	0.669	0.743	0.704
N3	N2	Validation	4	Full	0.707	0.723	0.826	0.686	0.75	0.637	0.684	0.66	0.653	0.775	0.709
N3	N2	Validation	4	Base	0.692	0.727	0.828	0.657	0.733	0.596	0.715	0.65	0.671	0.719	0.694
N3	N2	Validation	2	Full	0.613	0.83	0.63	0.598	0.613	0.56	0.513	0.535	0.654	0.773	0.709
N3	N2	Validation	2	Base	0.613	0.825	0.677	0.51	0.581	0.541	0.648	0.59	0.676	0.694	0.685
N2	N3	Test	All	Full	0.721	0.665	0.784	0.83	0.806	0.641	0.592	0.616	0.709	0.711	0.71
N2	N3	Test	All	Base	0.704	0.704	0.832	0.746	0.787	0.624	0.546	0.582	0.65	0.798	0.717
N2	N3	Test	FCO	Full	0.702	0.703	0.767	0.793	0.78	0.646	0.599	0.622	0.688	0.716	0.702
N2	N3	Test	FCO	Base	0.685	0.74	0.816	0.703	0.755	0.629	0.57	0.598	0.635	0.788	0.703
N2	N3	Test	6	Full	0.715	0.678	0.781	0.827	0.803	0.635	0.609	0.622	0.707	0.687	0.697
N2	N3	Test	6	Base	0.698	0.711	0.819	0.75	0.783	0.612	0.575	0.593	0.656	0.757	0.703
N2	N3	Test	4	Full	0.683	0.742	0.763	0.747	0.755	0.607	0.649	0.628	0.696	0.656	0.676
N2	N3	Test	4	Base	0.671	0.769	0.796	0.681	0.734	0.599	0.609	0.604	0.647	0.732	0.687

N2	N3	Test	2	Full	0.625	0.858	0.701	0.433	0.535	0.566	0.701	0.626	0.685	0.68	0.682
N2	N3	Test	2	Base	0.619	0.879	0.708	0.423	0.529	0.58	0.631	0.605	0.629	0.757	0.687
N2	N3	Validation	All	Full	0.706	0.703	0.78	0.839	0.808	0.587	0.544	0.565	0.68	0.646	0.663
N2	N3	Validation	All	Base	0.69	0.768	0.863	0.72	0.785	0.534	0.55	0.542	0.627	0.757	0.686
N2	N3	Validation	FCO	Full	0.678	0.758	0.758	0.797	0.777	0.59	0.532	0.56	0.642	0.659	0.651
N2	N3	Validation	FCO	Base	0.655	0.849	0.855	0.655	0.742	0.529	0.565	0.547	0.594	0.742	0.66
N2	N3	Validation	6	Full	0.701	0.71	0.782	0.836	0.808	0.588	0.565	0.576	0.668	0.624	0.645
N2	N3	Validation	6	Base	0.679	0.786	0.856	0.717	0.78	0.525	0.585	0.553	0.624	0.707	0.663
N2	N3	Validation	4	Full	0.631	0.866	0.781	0.664	0.717	0.505	0.649	0.568	0.639	0.563	0.599
N2	N3	Validation	4	Base	0.608	0.966	0.85	0.55	0.668	0.475	0.624	0.539	0.592	0.67	0.629
N2	N3	Validation	2	Full	0.564	0.949	0.685	0.387	0.494	0.5	0.687	0.579	0.617	0.59	0.603
N2	N3	Validation	2	Base	0.543	1.063	0.746	0.282	0.409	0.496	0.626	0.553	0.54	0.725	0.619

Table S6: Change in Accuracy by Sleep Stage

Node set	Feat set	Val. accuracy: train on N2, eval on N2	val acc N2:N3	val acc N3:N2	val acc N3:N3	rel_acc_change_N3N3_to_N2N2	rel_acc_change_N2N2_to_N3N2	rel_acc_change_N3N3_to_N2N3
All	Full	0.757	0.706	0.754	0.758	-0.001	-0.004	-0.069
6	Full	0.758	0.701	0.755	0.749	0.012	-0.004	-0.064
FCO	Full	0.727	0.678	0.729	0.734	-0.01	0.003	-0.076
4	Full	0.693	0.631	0.707	0.702	-0.013	0.02	-0.101
2	Full	0.635	0.564	0.613	0.594	0.069	-0.035	-0.051
All	Base	0.74	0.69	0.748	0.746	-0.008	0.011	-0.075
6	Base	0.736	0.679	0.746	0.738	-0.003	0.014	-0.08
FCO	Base	0.707	0.655	0.719	0.718	-0.015	0.017	-0.088
4	Base	0.67	0.608	0.692	0.684	-0.02	0.033	-0.111
2	Base	0.614	0.543	0.613	0.583	0.053	-0.002	-0.069

Table S7: SHAP laterality bias

SO Class	featset	Feature	Sleep Stage	nodeset	channel_pair	dset	Shap left	Shap right	Left - Right
F	all	delta power	N3	all	F3/F4	test	0.083	0.138	-0.04
F	all	delta power	N3	all	F3/F4	val	0.069	0.107	-0.05
F	all	delta power	N3	6	F3/F4	test	0.084	0.132	-0.04
F	all	delta power	N3	6	F3/F4	val	0.07	0.11	-0.10
F	all	delta power	N3	fco	F3/F4	test	0.08	0.178	-0.07
F	all	delta power	N3	fco	F3/F4	val	0.054	0.128	-0.08
F	all	delta power	N3	4	F3/F4	test	0.099	0.182	-0.10
F	all	delta power	N3	4	F3/F4	val	0.064	0.16	-0.13
F	all	delta power	N3	2	F3/F4	test	0.006	0.139	-0.17
F	all	delta power	N3	2	F3/F4	val	-0.057	0.112	0.14
F	all	delta power	N2	all	F3/F4	test	0.106	0.011	-0.01
F	all	delta power	N2	all	F3/F4	val	0.073	0.083	0.05
F	all	delta power	N2	6	F3/F4	test	0.114	0.063	0.02
F	all	delta power	N2	6	F3/F4	val	0.088	0.065	0.08
F	all	delta power	N2	fco	F3/F4	test	0.114	0.03	0.03
F	all	delta power	N2	fco	F3/F4	val	0.107	0.08	0.09
F	all	delta power	N2	4	F3/F4	test	0.143	0.057	0.08
F	all	delta power	N2	4	F3/F4	val	0.129	0.054	0.10
F	all	delta power	N2	2	F3/F4	test	0.04	-0.064	-0.11
F	all	delta power	N2	2	F3/F4	val	0.126	0.24	0.05
F	base	trough amp	N3	6	F3/F4	test	-0.13	-0.268	0.13
F	base	trough amp	N3	6	F3/F4	val	-0.076	-0.208	0.13
F	base	trough amp	N3	fco	F3/F4	test	-0.166	-0.294	0.10
F	base	trough amp	N3	fco	F3/F4	val	-0.119	-0.217	0.11
F	base	trough amp	N3	4	F3/F4	test	-0.194	-0.303	0.08
F	base	trough amp	N3	4	F3/F4	val	-0.134	-0.215	0.35
F	base	trough amp	N3	2	F3/F4	test	0.14	-0.207	0.28
F	base	trough amp	N3	2	F3/F4	val	0.12	-0.158	0.10
F	base	trough amp	N2	all	F3/F4	test	-0.14	-0.187	0.05
F	base	trough amp	N2	all	F3/F4	val	-0.118	-0.166	0.01
F	base	trough amp	N2	6	F3/F4	test	-0.161	-0.175	0.02
F	base	trough amp	N2	6	F3/F4	val	-0.138	-0.157	0.04
F	base	trough amp	N2	fco	F3/F4	test	-0.161	-0.198	0.04
F	base	trough amp	N2	fco	F3/F4	val	-0.129	-0.168	-0.02
F	base	trough amp	N2	4	F3/F4	test	-0.225	-0.201	0.04
F	base	trough amp	N2	4	F3/F4	val	-0.162	-0.198	-0.33
F	base	trough amp	N2	2	F3/F4	test	-0.123	0.209	-0.45
F	base	trough amp	N2	2	F3/F4	val	-0.212	0.239	0.41

F	base	trough amp	N3	all	F3/F4	test	-0.128	-0.27	0.14
F	base	trough amp	N3	all	F3/F4	val	-0.079	-0.215	0.14
G	all	delta power	N3	all	C3/C4	test	0.57	0.165	0.43
G	all	delta power	N3	all	C3/C4	val	0.56	0.128	0.39
G	all	delta power	N3	6	C3/C4	test	0.581	0.189	0.43
G	all	delta power	N3	6	C3/C4	val	0.567	0.138	0.37
G	all	delta power	N3	fco	C3/C4	test	0.732	0.362	0.33
G	all	delta power	N3	fco	C3/C4	val	0.682	0.353	0.36
G	all	delta power	N3	4	C3/C4	test	0.751	0.389	0.43
G	all	delta power	N3	4	C3/C4	val	0.753	0.322	-0.24
G	all	delta power	N2	all	C3/C4	test	0.173	0.653	-0.35
G	all	delta power	N2	all	C3/C4	val	0.23	0.581	-0.47
G	all	delta power	N2	6	C3/C4	test	0.176	0.646	-0.36
G	all	delta power	N2	6	C3/C4	val	0.195	0.551	-0.62
G	all	delta power	N2	fco	C3/C4	test	0.47	1.092	-0.49
G	all	delta power	N2	fco	C3/C4	val	0.478	0.969	-0.47
G	all	delta power	N2	4	C3/C4	test	0.537	1.011	-0.20
G	all	delta power	N2	4	C3/C4	val	0.556	0.755	-0.26
G	base	trough amp	N3	all	C3/C4	test	-0.663	-0.427	-0.22
G	base	trough amp	N3	all	C3/C4	val	-0.581	-0.36	-0.25
G	base	trough amp	N3	6	C3/C4	test	-0.651	-0.406	-0.20
G	base	trough amp	N3	6	C3/C4	val	-0.554	-0.357	-0.22
G	base	trough amp	N3	fco	C3/C4	test	-0.938	-0.715	-0.21
G	base	trough amp	N3	fco	C3/C4	val	-0.805	-0.592	-0.19
G	base	trough amp	N3	4	C3/C4	test	-0.964	-0.776	-0.17
G	base	trough amp	N3	4	C3/C4	val	-0.833	-0.659	-0.48
G	base	trough amp	N2	all	C3/C4	test	-0.663	-0.405	-0.35
G	base	trough amp	N2	all	C3/C4	val	-0.615	-0.265	-0.25
G	base	trough amp	N2	6	C3/C4	test	-0.647	-0.4	-0.22
G	base	trough amp	N2	6	C3/C4	val	-0.506	-0.29	-0.23
G	base	trough amp	N2	fco	C3/C4	test	-1.042	-0.815	-0.28
G	base	trough amp	N2	fco	C3/C4	val	-0.906	-0.628	-0.22
G	base	trough amp	N2	4	C3/C4	test	-1.152	-0.932	-0.20
G	base	trough amp	N2	4	C3/C4	val	-0.953	-0.751	0.19
L	all	delta power	N3	all	F3/F4	test	-0.088	-0.279	0.06
L	all	delta power	N3	all	F3/F4	val	-0.049	-0.106	0.20
L	all	delta power	N3	6	F3/F4	test	-0.094	-0.295	0.12
L	all	delta power	N3	6	F3/F4	val	-0.071	-0.186	0.19
L	all	delta power	N3	fco	F3/F4	test	-0.099	-0.285	0.10
L	all	delta power	N3	fco	F3/F4	val	-0.065	-0.169	0.21

L	all	delta power	N3	4	F3/F4	test	-0.099	-0.313	0.07
L	all	delta power	N3	4	F3/F4	val	-0.087	-0.155	0.27
L	all	delta power	N3	2	F3/F4	test	-0.139	-0.412	0.25
L	all	delta power	N3	2	F3/F4	val	-0.12	-0.374	-0.31
L	all	delta power	N2	all	F3/F4	test	-0.285	-0.126	-0.07
L	all	delta power	N2	all	F3/F4	val	-0.258	-0.187	-0.13
L	all	delta power	N2	6	F3/F4	test	-0.255	-0.127	-0.10
L	all	delta power	N2	6	F3/F4	val	-0.288	-0.192	-0.11
L	all	delta power	N2	fco	F3/F4	test	-0.269	-0.156	-0.06
L	all	delta power	N2	fco	F3/F4	val	-0.221	-0.162	-0.12
L	all	delta power	N2	4	F3/F4	test	-0.254	-0.135	-0.10
L	all	delta power	N2	4	F3/F4	val	-0.263	-0.166	-0.21
L	all	delta power	N2	2	F3/F4	test	-0.459	-0.248	-0.08
L	all	delta power	N2	2	F3/F4	val	-0.368	-0.288	-0.01
L	base	trough amp	N3	all	F3/F4	test	0.394	0.701	-0.26
L	base	trough amp	N3	all	F3/F4	val	0.299	0.562	-0.32
L	base	trough amp	N3	6	F3/F4	test	0.396	0.72	-0.25
L	base	trough amp	N3	6	F3/F4	val	0.315	0.563	-0.32
L	base	trough amp	N3	fco	F3/F4	test	0.374	0.691	-0.26
L	base	trough amp	N3	fco	F3/F4	val	0.288	0.551	-0.35
L	base	trough amp	N3	4	F3/F4	test	0.357	0.706	-0.29
L	base	trough amp	N3	4	F3/F4	val	0.273	0.56	-0.34
L	base	trough amp	N3	2	F3/F4	test	0.423	0.758	-0.26
L	base	trough amp	N3	2	F3/F4	val	0.325	0.583	-0.16
L	base	trough amp	N2	all	F3/F4	test	0.52	0.525	0.00
L	base	trough amp	N2	all	F3/F4	val	0.471	0.468	-0.05
L	base	trough amp	N2	6	F3/F4	test	0.495	0.545	-0.10
L	base	trough amp	N2	6	F3/F4	val	0.407	0.509	-0.02
L	base	trough amp	N2	fco	F3/F4	test	0.534	0.55	-0.07
L	base	trough amp	N2	fco	F3/F4	val	0.457	0.526	-0.04
L	base	trough amp	N2	4	F3/F4	test	0.498	0.537	-0.08
L	base	trough amp	N2	4	F3/F4	val	0.418	0.494	0.08
L	base	trough amp	N2	2	F3/F4	test	0.785	0.706	-0.06
L	base	trough amp	N2	2	F3/F4	val	0.582	0.637	0.00
